# Reliably Detecting Clinically Important Variants Requires Both Combined Variant Calls and Optimized Filtering Strategies

**DOI:** 10.1371/journal.pone.0143199

**Published:** 2015-11-23

**Authors:** Matthew A. Field, Vicky Cho, T. Daniel Andrews, Chris C. Goodnow

**Affiliations:** 1 Department of Immunology, John Curtin School of Medical Research, Australian National University, Canberra, ACT, Australia; 2 Australian Phenomics Facility, Australian National University, Canberra, ACT, Australia; 3 National Computational Infrastructure, Australian National University, Canberra, ACT, Australia; 4 Immunogenomics Group, Immunology Research Program, Garvan Institute of Medical Research, Darlinghurst, NSW, Australia; Mayo Clinic, UNITED STATES

## Abstract

A diversity of tools is available for identification of variants from genome sequence data. Given the current complexity of incorporating external software into a genome analysis infrastructure, a tendency exists to rely on the results from a single tool alone. The quality of the output variant calls is highly variable however, depending on factors such as sequence library quality as well as the choice of short-read aligner, variant caller, and variant caller filtering strategy. Here we present a two-part study first using the high quality ‘genome in a bottle’ reference set to demonstrate the significant impact the choice of aligner, variant caller, and variant caller filtering strategy has on overall variant call quality and further how certain variant callers outperform others with increased sample contamination, an important consideration when analyzing sequenced cancer samples. This analysis confirms previous work showing that combining variant calls of multiple tools results in the best quality resultant variant set, for either specificity or sensitivity, depending on whether the intersection or union, of all variant calls is used respectively. Second, we analyze a melanoma cell line derived from a control lymphocyte sample to determine whether software choices affect the detection of clinically important melanoma risk-factor variants finding that only one of the three such variants is unanimously detected under all conditions. Finally, we describe a cogent strategy for implementing a clinical variant detection pipeline; a strategy that requires careful software selection, variant caller filtering optimizing, and combined variant calls in order to effectively minimize false negative variants. While implementing such features represents an increase in complexity and computation the results offer indisputable improvements in data quality.

## Introduction

Rapid improvements in massively parallel sequencing technologies have dropped the cost of sequencing to the point where it is feasible to use patient sequence data in the clinic in order to identify both disease-causing and risk-factor variants that predispose patients to certain disease [1]. While data-specific computational algorithms aimed at deriving accurate data from these technologies have reached maturity [2], the routine use of genomic data for improving clinical diagnosis and treatment requires formalizing existing research methods into clinical best practices; the goal of recent initiatives like the CLARITY challenge [3]. In the interim, bioinformaticians are increasingly developing local, in-house production variant detection pipelines, the core of which typically consists of a highly customized workflow utilized by a relatively small local user-base, being developed out of necessity as few available software systems support the easy ‘pipelining’ of large biological datasets through multiple analytical tools to produce the desired end result. As a result, standardization of such pipelines is challenging largely due to the diversity of rapidly developing software tools being utilized, though some progress toward standardization efforts are beginning to emerge from the widespread use of the GATK software package [4].

The last several years has seen the creation of software frameworks for the management of bioinformatics pipelines which generally fall into two categories; easy to use GUI and web based approaches like GALAXY [5] and Taverna [6] compared to language specific frameworks such as GATK’s Queue (https://github.com/broadgsa/gatk/), BPipe [7] and Snakemake [8]. While these tools do address many of the key requirements of a high-throughput informatics systems, they typically anticipate that analysis will be linear and processive, an assumption which does not hold true in many instances. In addition, surprisingly few of these tools currently support large numbers of users, with the notable exception of GALAXY, which enjoys active support from a large broad-based community of users and developers (https://biostar.usegalaxy.org/). GALAXY and other web-based tools however, are not always the immediate choice for a high-volume production system due to potential challenges with transferring large volumes of data and the unavailability of highly specialized workflows.

Given the fast moving nature of both sequencing technologies and bioinformatic software development, successful and enduring informatics frameworks must remain flexible with regard to software selection, a central idea in the development of Bioconductor [9]. Such flexibility requires systems to routinely evaluate new software particularly in light of recent publications demonstrating low concordance levels between variant detection pipelines [10, 11]. Such differences arise not only due to software choices but also due to sequencing technology choices with studies demonstrating large differences in variant calls using different exome capture systems [12–14] and whole genome sequencing platforms [15, 16]. While the choice of sequencing technology and software is important, the internal parameters utilized for each algorithm are also important, particularly the filtering options employed by variant callers, a feature known to affect the overall variant call quality [17, 18]. Another important consideration for a framework is the ability to support testing software both in isolation as well as in various combinations (e.g. aligner / variant caller pairs) with previous studies showing the choice of short read aligner significantly impacts downstream variant calling, particularly for indels [19]. Further, with additional studies reporting both platform-specific [15, 20, 21] and software-specific [19] variants, it is clear that ideally a framework will support variant calls from multiple tools and sequencing platforms, particularly when avoiding false negative variants is the highest priority, as is the case when using variation data in a clinical context.

Assessing the quality of variant calls from any variant detection pipeline is greatly facilitated by the recent development of high quality reference data sets such as ‘genome in a bottle’ or GIAB [22]. Studies demonstrating low concordance levels amongst variant callers [23, 24] demonstrate the importance of software selection and highlight the need for standardized frameworks such as GCAT [25] which make it possible to assess variant calls relative to a set of validated high quality variants. While such frameworks are useful for accessing the results from a single processive analysis, tools such as BAYSIC [26] have shown that aggregating variants from multiple variant callers yields an overall improvements in both sensitivity and specificity compared to any individual tool, making it increasingly clear multiple variant callers are preferable in circumstances where either minimizing false positives or false negatives are crucial to a project. While many aspects of variant calling algorithms are similar, combining them leads to a substantial improvement in output quality justifying their use in parallel in certain circumstances. The utility of this approach is that through combining the relative sensitivities of different tools, a broader sensitivity is gained through taking the union of multiple calling methods. Similarly, performing variant calling through different algorithms in parallel, the quirks or systematic errors of a single tool may be overcome by taking the intersection of multiple approaches. It is for these reasons we have enabled combined variant calls in our in-house high throuhgput production system, yet, given these advantages, few specific workflows used in practice allow integrating tools in parallel in such a manner. This may be due to the increased computational load or due to structural limitations of workflow management tools.

In this work we first seek to demonstrate the significant impact the choice of aligner, variant caller, and variant caller filtering strategy has on both the number and quality of variant calls using high quality NA12878 genotype calls as a baseline (ftp://ftp-trace.ncbi.nih.gov/giab/ftp/data/NA12878/analysis/GIAB_integration/). Next, using the same data set, we replace increasing portions of the GIAB sequence data with non-variant reference data to determine the effectiveness of variant callers at increasing levels of simulated sample contamination. This is important given the increased use of sequenced cancer data in the clinic with cancer samples known to differ with regard to tumour purity [27], mutational heterogeneity [28], and subclonality [29]. Finally, using a melanoma cell line control sample, we demonstrate the impact the choice of software and filtering strategy has on the detection of clinically important melanoma risk-factor variants. For all analyses, the three different variant types assessed in this study (SNVs, small insertions, and small deletions) are analyzed independently to assess whether any single algorithm is superior to all others tested for all variant types. We conclude by presenting a cogent strategy for implementing a variant detection pipeline for clinical use; a pipeline focused on minimizing the total number of false negative variants.

## Materials and Methods

### Samples

#### GCAT Sample

The two FASTQ files ‘illumina-100bp-pe-exome-150x’ were downloaded from GCAT (http://www.bioplanet.com/gcat) and variant calls compared against GIAB high-confidence genotypes for NA12878 (ftp://ftp-trace.ncbi.nih.gov/giab/ftp/data/NA12878/analysis/GIAB_integration/). The sequence data consists of 45 million 100bp read pairs for estimated exome coverage of 150X.

#### Melanoma cell line control sample

Biospecimens were provided by members of the ABN-Oncology group, which is funded by the National Health and Medical Research Council. Sample C001 is a control lymphocyte derived sample from a female patient available for download at https://ccgapps.com.au/bpa-metadata/melanoma/sample/102.100.100.7688/ with the corresponding tumour sample (not used in this analysis) also available at https://ccgapps.com.au/bpa-metadata/melanoma/sample/102.100.100.7687/. For sequencing, the library construction was performed using TruSeq DNA Sample Preparation kits as per Illumina instructions. 1 μg of sample DNA was fragmented into 300–400 bp average insert size with 3’ or 5’ overhangs. End repair mix was then used to convert the fragmented DNA into blunt ends by removing the 3ʹ overhangs and the polymerase activity fills the 5’ overhang. The 3’ ends were then acetylated to add a single “A” nucleotide to the 3’ to reduce chimera formation. Ligate adapters were then used to attach adapters to the DNA fragments so they could be loaded into a flow cell and purified to remove unligated adapters to generate a final product with an insert size of 300–400 bp. PCR was then used to selectively enrich DNA fragments with adapter molecules at both ends for sequencing. Post amplification quality controls were performed using DNA High Sensitivity Labchips (Agilent Bioanalyzer). The libraries were then pooled and clustered using the iBOT and ready for sequencing. The 100 bp pair-end library was sequenced on a Hiseq2000 using a Truseq SBS V3-HS kit. The sequence data for C001 consists of 925 million 100bp read pairs for an estimated genome-wide coverage of 60X.

### System and Implementation

All analyses described was performed using an in-house production genomics analysis framework; a framework which bundles analytical processes as externally developed, compiled binary objects, driven by a custom Perl module layer that wraps individual tools. Each step in the workflow is driven at a scripting level and the command to these driver scripts and the associated parameters are defined within an XML configuration file—which allows customization, quick tool substitution, and straightforward change and extension of the workflow. Furthermore, archived workflows may be easily recreated with this static XML file from a particular analysis, along with the code repository revision number, allowing quick reproduction of archived analyses. Initially designed to detect SNVs in the exome sequence of progeny of C57BL/6 laboratory mice exposed to the spermatogonial point-mutagen *N*-ethyl-*N*-nitrosourea (ENU) [30], the expanded framework includes workflows for SNV/indel detection in human exomes and genomes, as well as custom multi-sample workflows to identify causal variation across sequenced human pedigrees [31] and paired tumour-normal analyses [32]. The total versioned code-base currently utilizes an ever-changing catalogue of open-source components combined with bespoke analysis tools–currently all linked via an underlying MySQL (http://www.mysql.com) tracking database (S1 Fig) designed for persistence, archiving and querying of summary results. A more detailed description of the design and implementation of the system can be found in supplementary data (S1 File).

### Expanded Software Assessment

**Fig 1:**
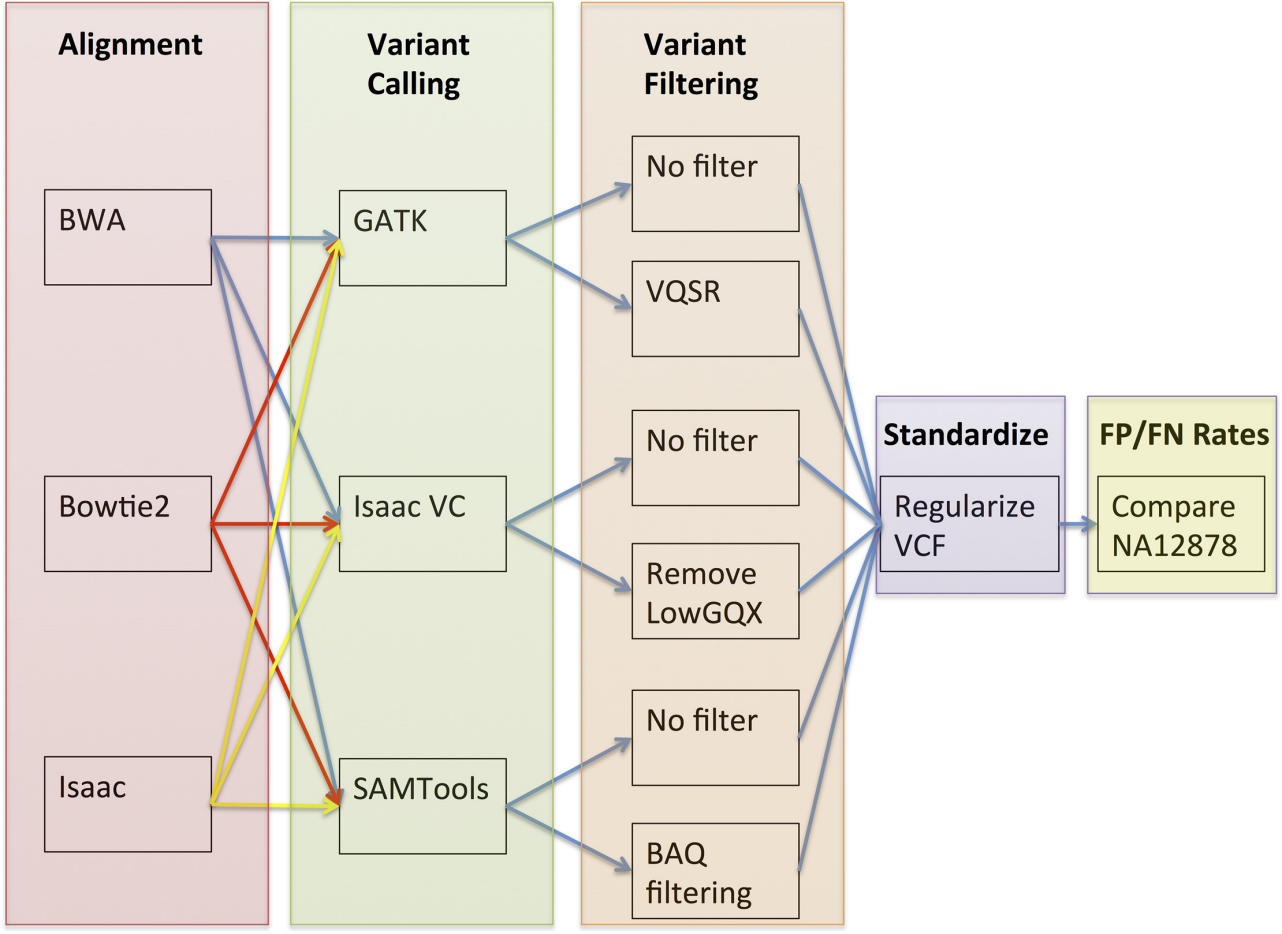
Analysis Workflow. BAM files from BWA, Isaac aligner, and Bowtie2 were paired with each of GATK, Isaac variant caller, and SAMTools run both with and without additional filtering (VQSR, BAQ, and LowGQX respectively). Output vcf files were regularized using custom code and variants from GIAB high quality regions taken forward to generate false positive and false negative rates.

The default variant calling workflow (S2 Fig) was expanded to pair three short read aligners (BWA [33], Bowtie 2 [34], and Isaac-aligner [35]) with three variant callers (GATK [4], Isaac-variant-caller [35], and SAMtools [36]), each of which was run with both with and without additional filtering (Fig 1). For all 18 possible aligner/variant caller pairs, software was run using either default options or as per suggestions in associated documentation (S1 Table). For each aligner, reads were aligned to the human reference genome (assembly GRCh37) and a sorted, indexed BAM file generated using SAMtools. Each BAM file was provided as input to each variant caller to generate a VCF file of unfiltered variant calls. To obtain filtered variant calls, GATK was run through variant quality score recalibration steps (VQSR) as documented at https://www.broadinstitute.org/gatk/guide/article?id=2805, SAMtools was run with and without full BAQ filtering and Isaac variant had all LowGQX annotated variants removed (defined as variants with a GQX score less than 30 or not present with GQX being the minimum of genotype quality score assuming variant and non-variant locus). Next, all variants with quality scores of less than 40 were removed and variants regularized using custom code and further divided into SNVs, insertions, and deletions lists. These lists were reduced to only variant contained in the high quality genotype GIAB regions and finally compared to the corresponding GIAB variant type to obtain false positive rates. False negative rates were estimated by taking all variants in the high confidence variant calling regions found to also overlap ENSEMBL v75 canonical transcripts. This reduction in variant search space was required to account for the fact that exome sequence data was utilized in this analysis.

Variants were classified as matching when the following criteria were met:

1. both variants are of the same types; either SNV, insertion, or deletion
2. both variants share the same start coordinate
3. both variants share the same end coordinate

To ensure consistent reporting of genomic coordinates for indels (an issue particularly problematic with indels falling in repetitive genomic sequences), vcf files are regularized with indels assigned the lowest genomic coordinate; for example a missing GC in a string of repeating GC’s is recorded as a deletion of the first GC encountered in the 5’ to 3’ direction within the larger repeat sequence range. Results are summarized for SNVs, deletions, and insertions (S2–S4 Tables respectively).

### Individual Software Assessment

To assess both the number and quality of variant calls for each aligner and variant caller in isolation, for each tool the union of all variants from all pairing was calculated and overlapped with GIAB variants and false positive and false negative rates calculated. For example, to assess GATK SNV calling efficiency, the union of SNVs called by GATK (paired with BWA, Bowtie2, or Isaac aligner input) was calculated and the merged list subsequently compared with high quality GIAB SNVs. Variant calls unique to a single software tool were also identified (referred to as ‘tool-specific variants’) and independently compared to GIAB variants to obtain false positive rates.

### Overall Software Concordance

**Fig 2:**
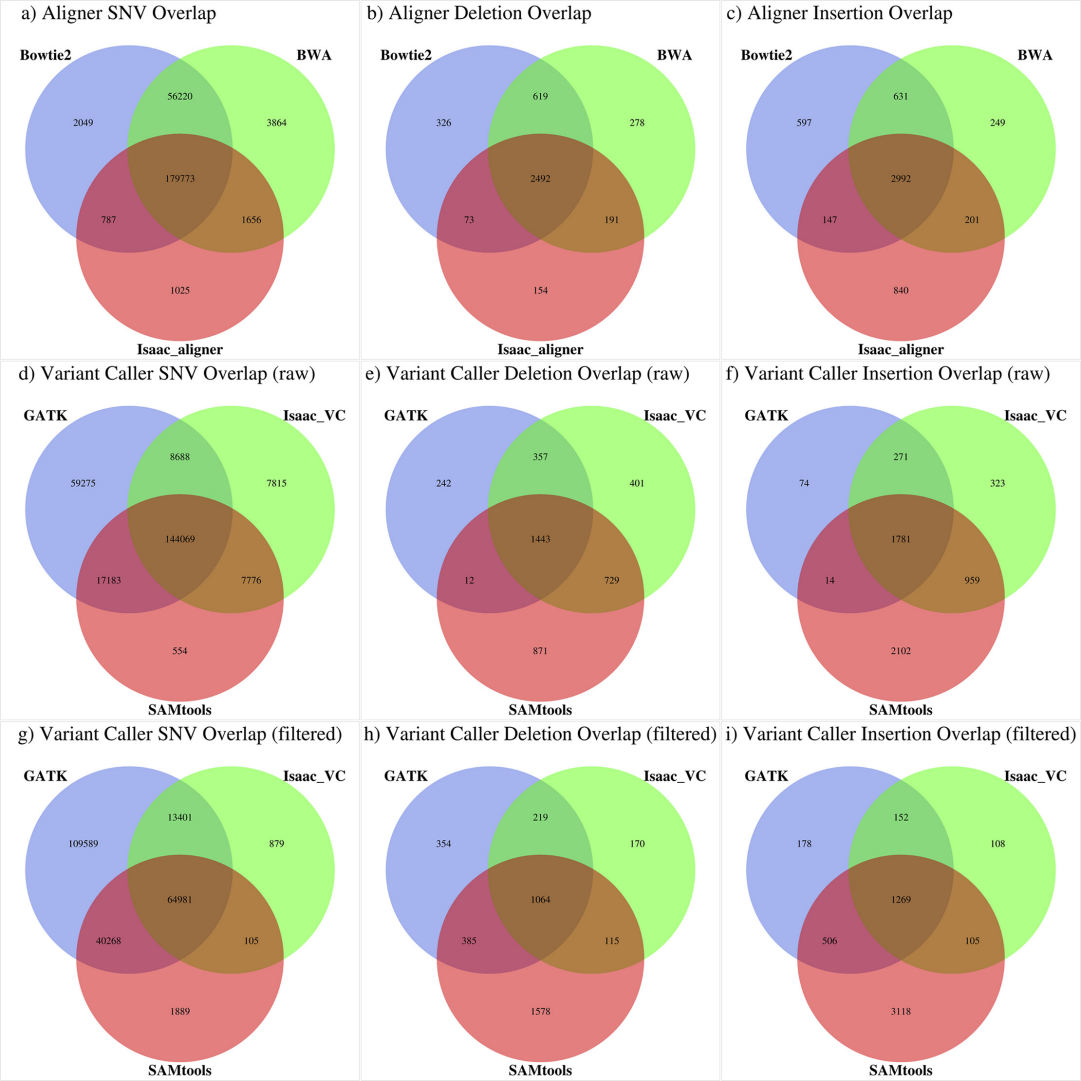
Software concordance Venn diagrams. Merged variant calls for each tool were overlapped with other tools of the same variety for each variant type. Aligners are compared in row 1, variant callers without filtering in row 2 and variant callers with filtering in row 3.

**Fig 3:**
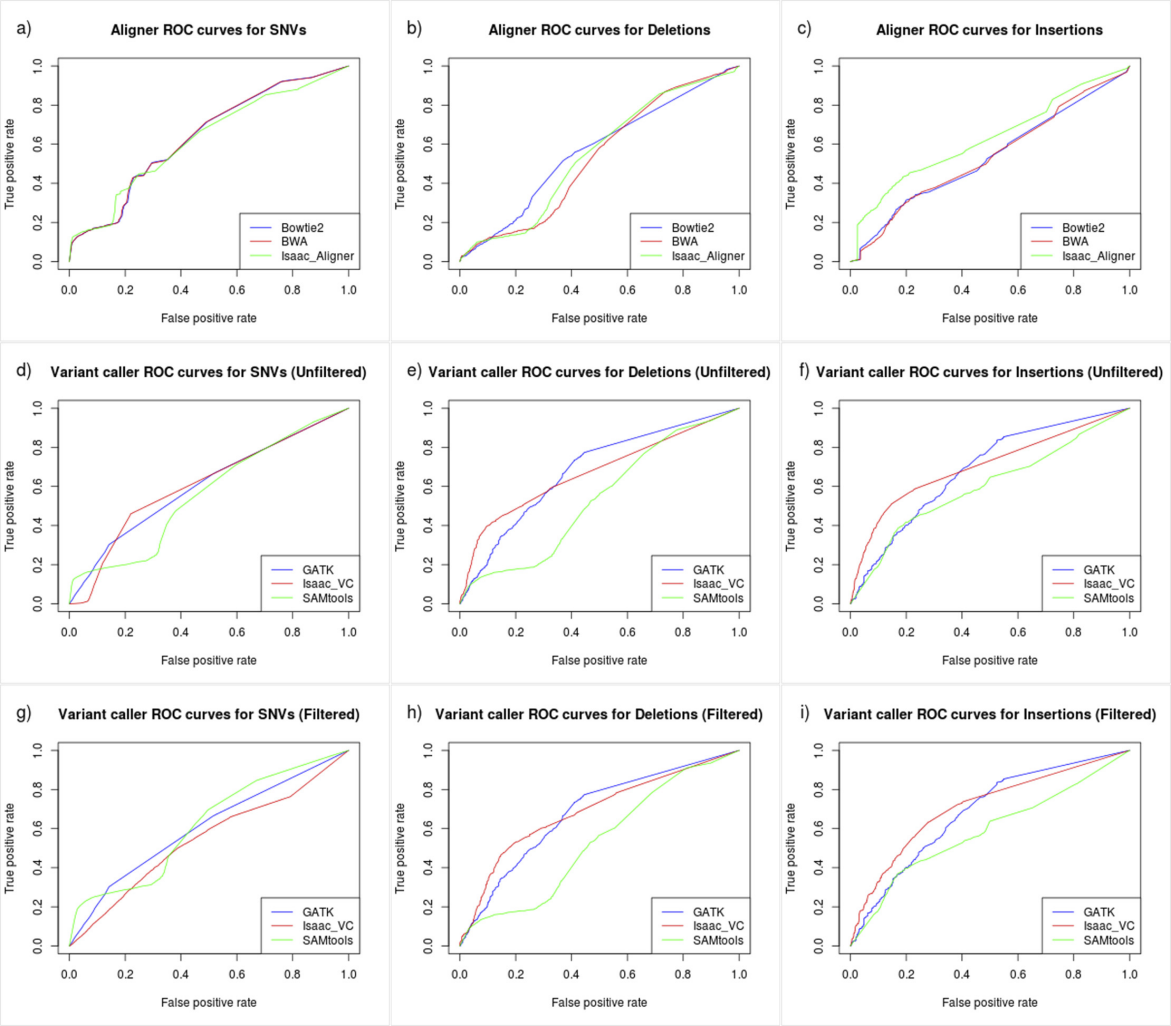
Software concordance ROC curves. Merged variant calls for each tool were calculated and ROC curves generated using the genome quality score. Aligners are compared in row 1, variant callers without filtering in row 2, and variant callers with filtering in row 3.

To measure the concordance of variant calls between the three aligners, the union of variants detected by each aligner was calculated and both Venn diagrams (Fig 2) and ROC curves plotted using genotype quality score (Fig 3) with the R packages VennDiagram [37] and ROCR [38] respectively. Similarly, variant caller concordance compared the output of GATK, Isaac variant caller, and SAMtools for both unfiltered and filtered variant calls. To assess how overall variant quality changed relative to the frequency of detection the number of times a variant was detected by all aligner / unfiltered variant caller pairs was calculated and divided into four categories; variants detected by at least 1 pair (union), variants detected by all 9 pairs (intersection), variants detected by 2–8 pairs, and variants detected by only 1 pair.

### Heterogeneous Sample Simulation

To determine the impact of sample heterogeneity on variant detection, increasing portions of GIAB sequence data was replaced with mutation-free reference genome reads generated using the SAMtools utility wgsim. For each simulated contamination level, the total sequence coverage level was kept constant at 150X with contamination levels of 0%, 25%, 50%, 75%, 90%, 95%, 98%, and 99% generated. At each level, reads were aligned to the reference genome with BWA and SNVs called with GATK, Isaac variant caller, and SAMtools with filtering applied. False negative and false positive levels were obtained by comparing variant calls to the GIAB high quality variant regions.

### Clinically Important Variants

To identify clinically important variants, all melanoma cell line control variants were overlapped with ClinVar (downloaded on October 24, 2014; [39]) and ClinVar entries classified as ‘pathogenic’ or ‘risk factor’ were identified with any melanoma risk factors variants prioritized.

## Results

### Individual Software Assessment

For each software tool, the union of all variants and tool-specific variants was calculated with false positive and false negative rates determined for SNVs (Table 1), deletions (Table 2), and insertions (Table 3). For each variant type, the false positive and false negative rates differed substantially with even greater differences observed for the tool-specific variants. For the aligners, BWA called the greatest number of total SNVs and deletions while Bowtie2 generated the most insertion calls. While Isaac aligner had fewer variant calls in general it had the lowest false positive rates for SNVs and deletions, with BWA having the lowest false positive rate for insertions. Considering false negative rates, BWA had the lowest rate for SNVs and deletions while Bowtie2 had the lowest rate for insertions. For the tool-specific variants, the results differed relative to variant type with BWA calling the most SNVs with the second lowest false positive rate, Bowtie2 calling the most deletions with the lowest false positive rate, and Isaac aligner calling the most insertions with the lowest false positive rate. For the unfiltered variant calls from the three variant callers, GATK called ~25% (59,275) more SNVs than either SAMtools or Isaac variant caller while SAMtools called the greatest number of insertions and deletions. SAMtools had the lowest false positive rate for SNVs while Isaac variant caller had the lowest false positive rate for insertions and deletions. For the false negative rate, SAMtools had the lowest rate for deletions, Isaac variant caller the lowest rate for insertions, and GATK the lowest rate for SNVs. Considering the tool-specific variants, SAMtools called the most insertions and deletions and GATK the most SNVs with GATK-specific variants having consistently low false positive rates, much lower than either SAMtools or Isaac variant caller specific variants. Finally, for the filtered variant calls, filtering uniformly increased the false negative rate and decreased the false positive rate for SNVs while the effect on indel calls was mixed. For indels only GATK filtering resulting in a decrease in the false positive rate with SAMtools and Isaac variant caller filtering yielding higher false positive rates.

**Table 1:** Total SNV calls and tool-specific SNV calls for each aligner and variant caller run with and without filtering.

Software	Filter	Total SNV Calls	False Positive Rate	False Negative Rate^a^	Tool-specific SNVs	Tool-specific FP Rate
Bowtie2	N/A	238829	8.44%	3.15%	2050	53.41%
bwa	N/A	241513	8.55%	2.96%	3860	39.27%
Isaac_align	N/A	183241	4.02%	3.31%	1030	36.12%
GATK	None	229215	7.43%	2.91%	59275	15.19%
GATK	VQSR	228239	7.06%	2.99%	N/A	N/A
Isaac_VC	None	168348	6.35%	4.27%	7815	32.50%
Isaac_VC	LowGQX	79366	4.56%	7.47%	N/A	N/A
Samtools	None	169582	6.12%	3.68%	554	86.64%
Samtools	BAQ	107243	3.84%	6.52%	N/A	N/A

The union of variant calls for each tool was calculated and false positive and false negative rates determined relative to high quality GIAB variants. Tool-specific calls were also calculated, defined as SNVs specific to a single tool.

^**a**^From bases overlap ENSEMBL v75 canonical transcripts.

**Table 2:** Total deletion calls and tool-specific deletion calls for each aligner and variant caller run with and without filtering.

Software	Filter	Total Deletion Calls	False Positive Rate	False Negative Rate^a^	Tool-specific Dels	Tool-specific FP Rate
Bowtie2	N/A	3617	33.26%	23.73%	379	29.29%
bwa	N/A	3676	30.88%	23.73%	252	46.03%
Isaac_align	N/A	2976	25.5%	27.12%	157	43.95%
GATK	None	2084	28.69%	27.12%	260	17.31%
GATK	VQSR	2049	27.57%	28.18%	N/A	N/A
Isaac_VC	None	2964	26.05%	23.73%	414	45.17%
Isaac_VC	LowGQX	1602	31.34%	35.59%	N/A	N/A
Samtools	None	3160	29.4%	27.12%	958	50.21%
Samtools	BAQ	3247	30.49%	27.12%	N/A	N/A

The union of variant calls for each tool was calculated and false positive and false negative rates determined relative to high quality GIAB variants. Tool-specific calls were also calculated, defined as deletions specific to a single tool.

^**a**^From bases overlap ENSEMBL v75 canonical transcripts.

**Table 3:** Total insertion calls and tool-specific insertion calls for each aligner and variant caller run with and without filtering.

Software	Filter	Total Insertion Calls	False Positive Rate	False Negative Rate^a^	Tool-specific Ins	Tool-specific FP Rate
Bowtie2	N/A	4367	27.11%	22.39%	565	35.04%
bwa	N/A	4073	20.94%	31.34%	215	61.40%
Isaac_align	N/A	4180	31.27%	31.34%	843	14.95%
GATK	None	2140	20.56%	35.82%	74	14.86%
GATK	VQSR	2105	19.62%	37.31%	N/A	N/A
Isaac_VC	None	3334	19.26%	25.37%	323	56.97%
Isaac_VC	LowGQX	1634	23.32%	34.33%	N/A	N/A
Samtools	None	4856	34.1%	22.39%	2102	41.29%
Samtools	BAQ	4998	34.87%	20.90%	N/A	N/A

The union of variant calls for each tool was calculated and false positive and false negative rates determined relative to high quality GIAB variants. Tool-specific calls were also calculated, defined as insertions specific to a single tool.

^**a**^From bases overlap ENSEMBL v75 canonical transcripts.

### Overall Software Concordance

To assess the concordance of variant calls generated by the alignments from BWA, Bowtie2, and Isaac aligner, the merged variant lists for each aligner were overlapped to generate Venn diagrams with a similar approach taken to measure concordance levels between both filtered and unfiltered variant calls generated by GATK, Isaac variant caller, and SAMTools (Fig 2). For the three aligners, 73.26% of SNV calls were unanimously detected compared to only 58.71% of unfiltered SNVs from the three variant callers. For deletions, the three aligners shared 55.89% of all deletion calls compared to only 35.58% of unfiltered deletions from the three variant callers. Lastly for insertions, the three aligners shared 52.89% of all insertion calls compared to only 32.24% of unfiltered insertions from the three variant callers. In addition to Venn diagrams, ROC plots were generated for each variant type using genotype quality as input (Fig 3).

### Variant Type Comparison

Variants were segregated based on the frequency of detection for each variant types (Table 4). Overall, SNV calls exhibiting the greatest concordance levels with 43.45% of the total 245,360 SNVs detected by all pairs, 54.20% detected by 2–8 pairs, and 2.35% detected by a single pair. Deletions had the next highest concordance levels with 27.61% of the total 4194 deletions calls detected by all pairs, 55.46% detected by 2–8 pairs, and 16.93% unique to a single pair. Lastly, insertions exhibited the lowest concordance levels with only 25.62% of the total 5524 insertions detected by all pairs, 47.28% detected by 2–8 pairs, and 27.10% unique to a single pair.

**Table 4:** Variant calls grouped by frequency of detection.

Variant Type	Number of times variant detected (out of 9 total software pairs)	Total Variant Calls	False Positive Rate^a^	False Negative Rate
SNV	9 (Intersection)	106594	3.29%	6.78%
	> = 1 (Union)	245360	9.09%	2.90%
	2–8	132989	12.18%	N/A
	1	5777	46.43%	N/A
Deletion	9 (Intersection)	1158	14.68%	35.59%
	> = 1 (Union)	4194	35.13%	23.72%
	2–8	2326	36.54%	N/A
	1	710	63.80%	N/A
Insertion	9 (Intersection)	1415	12.16%	46.26%
	> = 1 (Union)	5524	35.36%	19.40%
	2–8	2612	26.03%	N/A
	1	1497	73.55%	N/A

Unfiltered variants were grouped based on the frequency of detection within nine possible aligner/variant caller pairs and segregated into four bins; variants in all 9 pairs, variants in at least 1 pair, variants in 2–8 pairs, and variants unique to 1 pair.

^**a**^From bases overlap ENSEMBL v75 canonical transcripts.

Considering the union of all variant calls results in 245,360 unique SNV calls, 5524 unique insertion calls, and 4194 unique deletion calls with an average of 205,115 SNVs, 3787 insertions, and 3055 deletion calls per aligner/variant caller combination. Within these variant lists, SNVs had the lowest false positive and false negative rates (9.09% and 2.90% respectively), followed by deletions (35.13% FP and 23.72% FN), and insertions (35.36% FP and 19.40% FN). Considering the intersection of all variants (i.e. only unanimously called variants) generated 106,594 unique SNV calls, 1415 unique insertion calls, and 1158 unique deletion calls, all of which has lower false positive rates and higher false negative rates as expected. The false positive rate was 3.29% for SNVs, 14.68% for deletions, and 12.16% for insertions and the false negative rate was 6.78% for SNVs, 35.59% for deletions, and 42.26% for insertions. Finally, the variants unique to a single pair contained large numbers of false positives for SNVs (46.43% FP), deletions (63.80% FP), and insertions (73.55% FP).

### Heterogeneous Sample Simulation

Using BWA alignments filtered SNV lists for GATK, Isaac variant caller, and SAMtools were generated at simulated contamination levels of 0%, 25%, 50%, 75%, 90%, 95%, 98%, and 99% (Table 5). Each variant caller detected substantially fewer variants as contamination levels increased resulting in increasing false negative rates. The increase in the false negative rate was not consistent across all variant callers however, with GATK still able to detect 60% of all SNVs using only 10% of the original GIAB data compared to only 45% for Isaac variant caller and 35% for SAMtools. In general, GATK coped better with higher contamination levels than either SAMtools or Isaac variant caller with similar patterns observed for both deletions and insertions.

**Table 5:** SNV calls for GIAB data at simulated contamination levels.

Variant Caller	Simulated Contamination Level	Variant Calls	False Positive Rate^a^	False Negative Rate
GATK	0%	228239	6.62%	3.23%
	25%	163041	6.22%	3.76%
	50%	113263	5.50%	5.54%
	75%	69416	4.77%	13.45%
	90%	34018	4.13%	40.74%
	95%	17321	4.08%	67.55%
	98%	5662	4.06%	88.87%
	99%	1696	4.25%	96.46%
Isaac VC	0%	79366	4.51%	7.90%
	25%	73259	4.26%	12.84%
	50%	61458	4.01%	18.56%
	75%	43091	3.90%	31.59%
	90%	23146	3.67%	54.76%
	95%	10888	3.77%	76.29%
	98%	3246	4.13%	92.60%
	99%	887	5.41%	97.96%
SAMtools	0%	106332	3.78%	6.39%
	25%	83337	3.66%	9.17%
	50%	63382	3.46%	14.76%
	75%	39853	3.29%	30.92%
	90%	16660	3.16%	64.83%
	95%	6365	3.03%	85.64%
	98%	1534	3.46%	96.45%
	99%	371	6.47%	99.18%

BWA alignments were used to generate filtered SNV lists for GATK, Isaac variant caller, and SAMtools at simulated contamination levels of 0%, 25%, 50%, 75%, 90%, 95%, 98%, and 99%. Variant lists were overlapped to GIAB high quality variants to determine false positive and false negative rates.

^**a**^From bases overlap ENSEMBL v75 canonical transcripts.

### Clinically Important Variants

All variants calls from melanoma cell line control C001 were overlapped to ClinVar yielded 2266 matching SNVs and 9 matching deletions, all but four of which corresponded to existing dbSNP entries. Of the 2266 SNV calls, 1894 were unanimously detected (83.6%), with the remaining 372 (16.4%) missed by at least one software pair. From the ClinVar annotations, three variants were annotated as known melanoma risk factors with only one of such variants unanimously detected by all software combinations (Table 6).

**Table 6:** Melanoma cell line control variant calls overlapping annotated ClinVar melanoma risk factors.

GRCh37 Coordinate (dbSNP)	dbSNP id	Missing Aligner / Variant Caller Pair (F = filtered, U = unfiltered)	ClinVar Annotation
5:33951693	rs16891982	None	Malignant melanoma of skin
11:89017961	rs1126809	Bowtie2/Isaac_vc (F)	Increased risk of cutaneous melanoma
14:104165753	rs861539	Isaac_aligner/GATK (U, F) Isaac_aligner/Isaac_vc (U, F) Isaac_aligner/SAMtools (U, F)	Increased risk of cutaneous melanoma

Variant calls from melanoma cell line C001 were overlapped to ClinVar and all annotated melanoma risk factors examined. Software pairs failing to detect these variants are reported in column 3 with variants listed as unfiltered (U) or filtered (F) to reflect whether variant caller filtering was applied.

## Discussion

Ongoing efforts to characterize genetic variants for clinical action [40] and the ever-increasing number of previously characterized variants routinely being used for decisions in the clinic [41, 42] illustrate the potential importance of a single variant. From the analysis of the melanoma cell line control, we identified three important melanoma risk factor variants, two of which were not detected unanimously under all software conditions (Table 6). One missed risk factor variant, rs861539, was not detected by Isaac aligner when paired with any of the three variant callers (SAMtools assigned a failing variant score of 11, Isaac variant caller assigned a failing variant score of 8, and GATK did not flag the base as variant) highlighting the danger of relying on a single tool for any analysis step. The other missed risk factor variant, rs1126809, was missed by a single software combination (Bowtie2 and Isaac variant caller with filtering applied) as a result of the variant being annotated as low quality, illustrating the danger of applying aggressive filtering when considering variation data in a clinical context. While these two variants are significant in this instance, broader analysis of the GIAB data set showed that all aligners and variant callers assessed in this study fail to detect true variants that are detected by other algorithms, implying clinically important variants may be missed even when the top performing software is utilized. Given the importance of detecting clinically important variants accurately, the utility of first optimizing filtering strategies for individual algorithms and then combining variant calls from multiple variant callers is apparent as the increased output quality quickly justifies any increased computation.

Using the GIAB reference data, we compared the false positive and false negative rates of three short-read aligners paired with three variant callers run both with and without variant caller filtering for SNVs, deletions, and insertions (S2–S4 Tables). Here we confirmed previous work that the choice of aligner, variant caller, and variant caller filtering affects both the number of variants detected and their subsequent quality [11, 21, 23]. To better assess the performance of each individual algorithm in isolation the union of all variants generated by each tool was calculated for SNVs (Table 1), deletions (Table 2), and insertions (Table 3). While each algorithm tested caused an observable effect on variant call quantity and quality, this effect differed with regard to the type of variant, the overall false positive and false negative rates, and the quality of the tool-specific variants. For total variant calls, the variant caller choice had a greater impact than the aligner choice with 36% more SNVs called by GATK than Isaac variant caller, 51% more deletions called by SAMtools than GATK, and 127% more insertions called by SAMtools than GATK. While the choice of aligner had less impact than the choice of variant caller on total variant number, the effect was still significant with 32% more SNVs called by BWA than Isaac aligner, 24% more deletions called by BWA than Isaac aligner, and 7% more insertions called by Bowtie2 than BWA. The false positive rate also differed significantly ranging for SNVs from 3.84% for SAMtools to 8.55% for BWA, for deletions from 25.5% for Isaac aligner to 33.26% for Bowtie2, and for insertions from 19.26% for Isaac variant caller to 34.87% for SAMtools. Similarly, the false negative rate varied significantly ranging for SNVs from 2.91% for GATK to 7.47% for Isaac variant caller, for deletions from 23.73% for BWA and Bowtie2 to 35.59% for Isaac variant caller, and for insertions from 20.90% for SAMtools to 37.31% for GATK. When considering all variants called the range in quality is large, however the range is even greater when considering tool-specific variants. Tool-specific variants represent an important variant subset as they serve to highlight differences amongst the individual algorithm, with such variants being important to consider particularly when minimizing false calls is a priority. For example, there are 59,275 GATK-specific SNVs of which almost 85% are true variants meaning these will be missed unless GATK is utilized for SNV calling. While GATK seems an excellent choice for SNV detection, it calls substantially less tool-specific indels than SAMtools meaning no single algorithm is optimal for minimizing false calls across all variant types. Such results demonstrate the utility of tool-specific variants for making informed decisions about software selection; for example the consistently low false positive rate of ~15% for all types of GATK-specific variants led us to incorporate it into our production system. Collectively these results illustrate the significant and often-unpredictable effects the choice of aligner and variant caller has on variant call quality and highlights the importance of ongoing software appraisal and optimization.

Another important factor known to affect variant call quality is the filtering strategy applied by each of the variant caller software suites [17, 18]. Each variant caller employs a distinctive strategy for filtering; GATK uses variant quality score recalibration (VQSR), SAMtools uses BAQ filtering, and Isaac aligner annotates questionable variants having low scoring genotype quality as ‘lowGQX’. While these filtering strategies differ significantly algorithmically, they share the common goal of trying to remove false positive variants from the original variant lists. In our analysis, applying these filters mostly resulted in a reduced number of total calls as expected, with the exception of BAQ filtering which resulted in a slight increase in the number of indel calls as might be expected given it’s focus on removing false positive SNV calls around candidate indels. Interestingly, only GATK VQSR filtering reduced the false positive rate and increased the false negative rate across all variant types with both SAMtools and Isaac variant caller filtering yielding increased false positive rates for indels indicating potential issues with their respective indel filtering strategies. While the reduction in false positive rate is important, from a clinical context it is important to note that all filtering strategies removes true variants; ranging from as few as 73 true positive SNVs with GATK to the extreme case of the Isaac variant caller filtering removing over 100,000 true positive SNVs. Overall, these results highlight the large effect software choices make in both the precision and recall of variants.

In our analysis, no aligner / variant caller pair outperformed all other pairs across all variant types, illustrating the importance of selecting software specific to each variant type. In general, SNV calls yielded significantly lower false positive and false negative rates compared to indel calls with unanimously detected SNVs having FP/FN rates of 3.29% and 6.78% compared to 14.68% and 35.59% for deletions and 12.16% and 46.26% for insertions (Table 4). This difference is even more apparent when considering the union of all SNV calls with FP/FN rates of 9.09% and 2.90% compared to 35.13% and 23.72% for deletions and 35.36% and 19.40% for insertions. The higher false positive rate of indel calls might be explained if the GIAB reference set was under-reporting indels however this seems unlikely with GIAB reporting a SNV to indel ratio of 6:1 (2.89 million SNVs and ~465,000 million indels), a lower ratio than the 10:1 estimated by the 1000 genomes project [43]. A more likely explanation is that indels are scarcer than SNVs and more difficult to detect due to challenges of aligning short reads around indels [44] meaning the distinct patterns in alignments of short-read data requires distinct workflows for SNV and indel detection [35]. A final possibility is that the variant callers assessed in this analysis do not employ the most up to date methodologies for indel detection with new tools such as Scalpel [45] and ABRA [46] utilizing microassembly in the detection of indels. Such tools are reporting lower false positive rates than existing software and likely do offer improvements in the overall quality of indel calls. Regardless, with variant detection software rapidly improving, the need to select software specific to each variant type is clear.

Another increasingly common application utilizing variation data in the clinic is the use of cancer samples as a way of advancing personalized treatment of cancer [47]. Working with cancer samples has additional complications however, with factors such as sample contamination known to be a problem [27]. To measure the effect of contamination on variant detection, an additional analysis was undertaken where GIAB sequence data was replaced with increasing levels of non-variant reference data to simulate increasing levels of contamination (Table 5).

For simplicity, only the filtered SNV calls from GATK, Isaac variant caller, and SAMtools were assessed and as expected, we observe large increases in false negative rates as the contamination level increases. The ability to cope with contamination differed for the three variant callers however with GATK outperforming the others significantly; for example at 50% contamination levels GATK had a false negative rate of only 5.54% compared to 14.76% for SAMtools and 18.56% for Isaac variant caller. These results suggest additional considerations are required when analyzing samples likely to suffer from contamination, as is often the case for cancer samples. While this study focused on the detection of germ line variation, the nature of variation in cancer is fundamentally different from non-cancer with much of the current thinking based on the understanding that a clone accumulates somatically acquired mutations that ultimately leads to malignant transformation [48], with inherited germ line mutations thought to be important in only 5–10% of cancers [49]. Somatic mutation detection is not addressed in this study, however the design of this study could be easily applied to assess software such as MuSiC [50] or MuTect [51], software specifically designed to detect somatic mutations in paired samples.

While we have demonstrated the impact the choice of software, filtering strategy, and combined variant calls have on resultant variant calls, differences in sample and library preparation as well as coverage levels are also known to significantly affect variant calling [52]. For sample preparation, differences in input DNA amount, sample age, and sample preservation method are known to be important as are library issues such as PCR amplification errors, primer biases, chimeric reads, and barcode/adaptor errors. Depth of coverage has also been shown to have a large impact on variant detection [53] and is of particular importance when considering contaminated samples such as tumors, with additional coverage typically required to compensate for contamination. While in this study it is not feasible to exhaustively examine the impact of all such factors on variant detection, the sequencing of larger and larger number of samples will allow us to begin to understand the effect of such factors on variant detection.

Finally, we present a cogent strategy for creating a variant detection pipeline for clinical use that focuses on minimizing the total number of false negative variants from the onset. For all workflows, a single aligner is sufficient with BWA generating the greatest number of both SNV and deletion calls and only 7% less insertion calls than Bowtie2. In this study, we show the single most effective measure for minimizing false negative variants is combining the results from multiple variant callers. Implementing this approach requires running multiple variant callers in parallel and combining the results to take forward for analysis, an approach already implemented by tools such as BAYSIC [26]. While combined variant calls is the ideal strategy, it must be acknowledged that computational resources are often limited in which case it is preferable to run BWA paired with GATK due to the low false negative rates, particularly for SNVs and deletions. Finally, when sample contamination is likely to be an issue (as in cancer samples) GATK should be utilized as it outperforms Isaac variant caller and SAMtools at increasing contamination levels. While the strategy presented represents the most effective use of the tools assessed in this study, it is important to routinely reassess both new software and new versions of existing software to remain current in this fast moving field. Doing this routinely requires that any framework designed to support clinical usage of variation information must be able to easily run and benchmark a wide variety of software. Further, such a framework must be able to run multiple variant callers and combine their output, a feature missing in almost all modern pipeline frameworks.

## Conclusion

Analysis of the GIAB reference dataset shows that the choice of aligner, variant caller, and variant caller filtering strategy significantly affects the quality of variant calls and that true variants can be missed by individual software or removed during variant caller filtering. Analysis of the melanoma cell line control shows only one of three melanoma risk factor variants is detected unanimously with one variant missed completely by a single software tool and the other variant removed during variant caller filtering. These results demonstrate the importance of developing a strategy based on reducing false negative variants when utilizing variation data in a clinical context; a strategy that requires careful software selection, variant caller filtering optimization, and combined variant calls from multiple variant callers.

## Supporting Information

S1 Fig Tracking database schema. Tracking database schema generated using MySQL Workbench. The database records all sample metadata, sequence data information, and the analysis steps performed. Any previous analysis can be completely reproduced solely from the information contained in the database.
(TIFF)

S2 Fig Default pipeline workflow. Default workflow for the production in-house pipeline. When new data is received the metadata is parsed to determine whether the sample is new and if so, what type of sample it is with available options for single human, single mouse, human pedigree, or human cancer. If new data is added to an existing sample it is linked to the original data and a new analysis run commences.
(TIFF)

S1 File Detailed Variant Detection Workflow. Detailed description of design and implementation of high-throughput in-house variant detection pipeline.
(DOCX)

S1 Table Short read aligner and variant caller versions and commands. All short read aligners and variant callers commands utilized in our example. The commands listed match the exact commands run with the exception of the shortening of file names. The commands were chosen by either following documentation suggestions, or else by using default options.
(DOCX)

S2 Table SNV call overlaps with GIAB. SNV stats from all eighteen possible software combinations derived from the pairing of the three aligners with each of the three variant callers run both with and without filtering. SNVs were overlapped to GIAB SNVs and false positive and false negative rates calculated.
(DOCX)

S3 Table Deletion call overlaps with GIAB. Deletion stats from all eighteen possible software combinations derived from the pairing of the three aligners with each of the three variant callers run both with and without filtering. Deletions were overlapped to GIAB deletions and false positive and false negative rates calculated.
(DOCX)

S4 Table Insertion call overlaps with GIAB. Insertion stats from all eighteen possible software combinations derived from the pairing of the three aligners with each of the three variant callers run both with and without filtering. Insertions were overlapped to GIAB insertions and false positive and false negative rates calculated.
(DOCX)
