# Supplementary figures and images for: Reliably Detecting Clinically Important Variants Requires Both Combined Variant Calls and Optimized Filtering Strategies
Source: PLoS One. 2015 Nov 23;10(11):e0143199. doi: 10.1371/journal.pone.0143199 (PMC4658170; doi:10.1371/journal.pone.0143199)

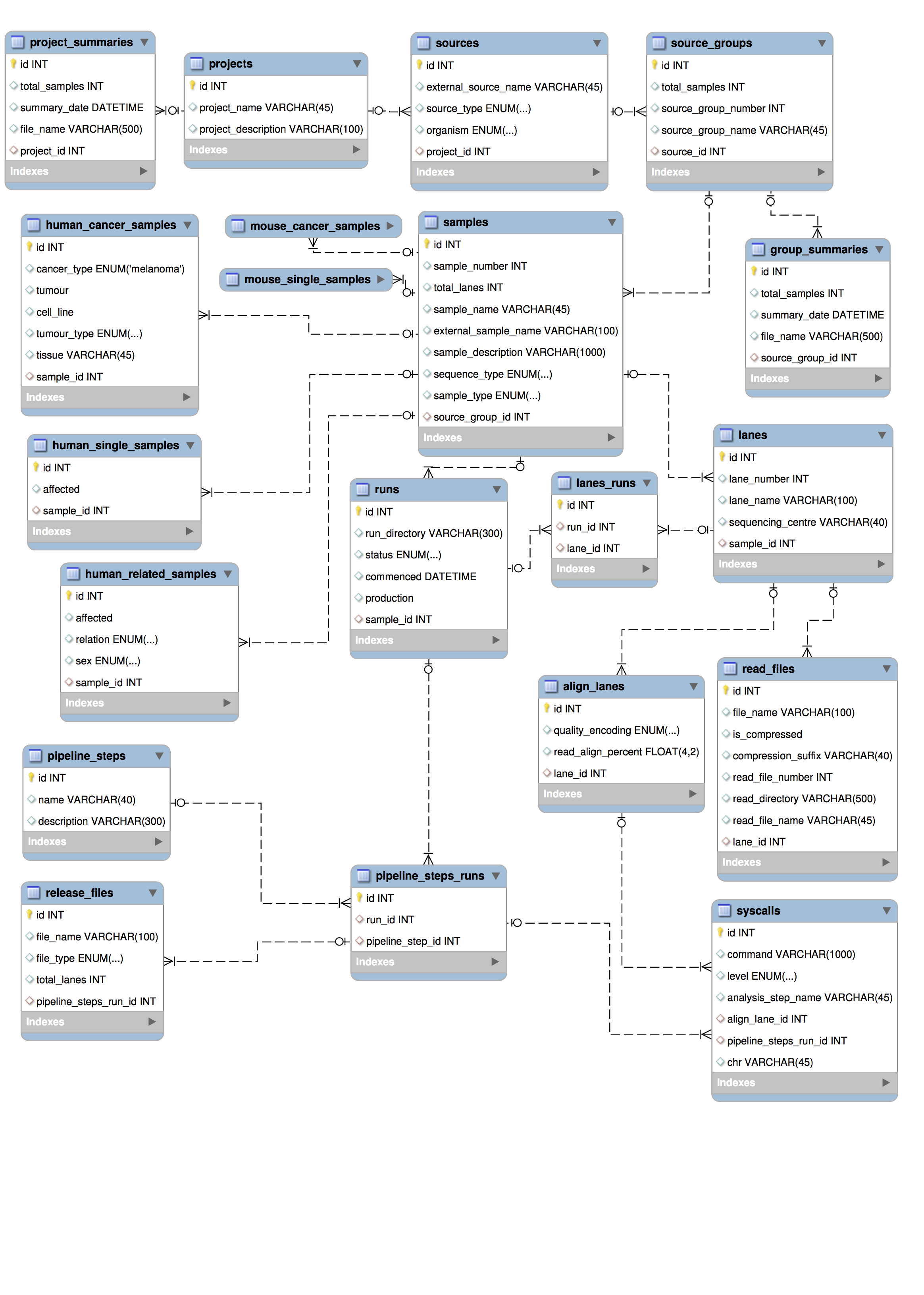

Supplement: S1 Fig — Tracking database schema generated using MySQL Workbench. The database records all sample metadata, sequence data information, and the analysis steps performed. Any previous analysis can be completely reproduced solely from the information contained in the database. (TIFF) [file pone.0143199.s001.tiff]

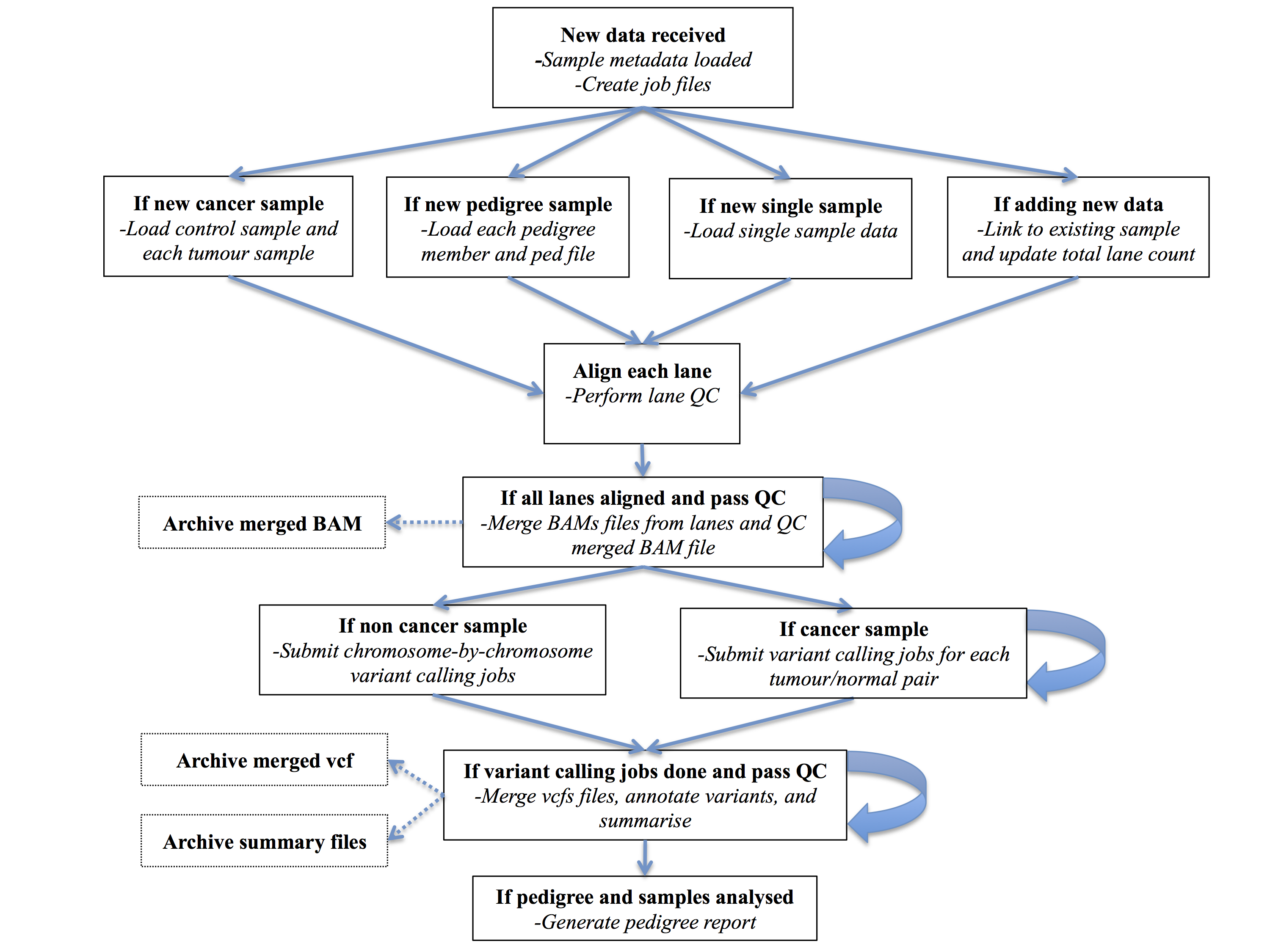

Supplement: S2 Fig — Default workflow for the production in-house pipeline. When new data is received the metadata is parsed to determine whether the sample is new and if so, what type of sample it is with available options for single human, single mouse, human pedigree, or human cancer. If new data is added to an existing sample it is linked to the original data and a new analysis run commences. (TIFF) [file pone.0143199.s002.tiff]
