# Supplementary material for: Reliably Detecting Clinically Important Variants Requires Both Combined Variant Calls and Optimized Filtering Strategies
Source: PLoS One. 2015 Nov 23;10(11):e0143199. doi: 10.1371/journal.pone.0143199 (PMC4658170; doi:10.1371/journal.pone.0143199)
Supplement: S1 File — Detailed description of design and implementation of high-throughput in-house variant detection pipeline. (DOCX) [file pone.0143199.s003.docx]

**S1 File. Detailed Variant Detection Workflow**

Sequence data for each sample is initially copied to a staging area on raijin (<http://nci.org.au/nci-systems/national-facility/peak-system/raijin/>) at the National Computational Infrastructure, the 38^th^ largest supercomputer in the world (<http://www.top500.org/>). Metadata for each sample is loaded from an external sample tracking database and read directories created automatically with all sequence data copied from the staging area to the read directory using regular expressions to determine the destination for each file. Next, sample metadata is loaded into a master configuration file containing information such the sample project, type of sample (cancer, pedigree, single, mouse, etc), read directory, and other relevant metadata specific to each sample. A cronjob running on the headnode of raijin checks for changes to the master config file and when new samples are identified the metadata is loaded into an internal tracking database (**Fig S1**) with all necessary HPC job files created simultaneously (similar to a tool like COSMOS (1)) and the initial BWA alignment jobs submitted (2). In order to reduce the overall run time multiple samples are analyzed in parallel, making the sole-limiting factor the amount of available compute resources. If the sample contains more than one lane of sequence data each lane is analyzed independently which offers two main advantages; lane-specific QC (which frequently serves in preventing corrupt lanes being merged with otherwise uncorrupted BAM files) as well as allowing new lanes to be added to existing samples at a later date if higher coverage levels are desired. To generate a BAM files, repetitively aligned reads are filtered out (identified by the XT:A:R tag) and a sorted BAM file generated using SAMtools (3). Alignment statistics are generated for each BAM file using the samtools flagstat command and only lanes with 90% or more of the reads aligning pass lane QC. When all individual lanes for a sample have passed QC, BAM files are merged into a single BAM file and run through samtools (3) rmdup to remove candidate PCR duplicates. Variants are next called using either samtools/bcftools (4) or GATK (5) best practices. Jobs are split up into chromosome-by-chromosome jobs (using a map-reduce like approach (6)), which run independently while a cronjob running on the headnode detecting when all jobs are complete and proceeds to merge the chromosome-by-chromosome results and submit the next job to run. Variants are annotated using ENSEMBL variant effect predictor (VEP) (7) and overlapped with ENSEMBL canonical transcripts and splice site variants, defined as the 10bp either side of a coding exon. Additional annotations not available from standard annotation tools are next added, annotations such as ExAC (<http://exac.broadinstitute.org/about>) or mouse phenotype information from MGI (<http://www.informatics.jax.org/>). Lastly, variant reports for SNVs and indels are generated and prioritized by several measures such as novel or low frequency variants, nonsense and missense mutations, high polyphen2 (8) and low SIFT (9) scores amongst others. Numerous custom filtering options to reduce the variant search space with filters based on genomic region, gene name, population allele frequencies, and polyphen/SIFT score are also available.

For running individual system commands, a generic framework was developed to reflect the fact that any large workflow can be broken down into smaller analysis components and ultimately individual system commands. To run a single analysis step, the wrapper first reads the sample’s configuration file to retrieve relevant step information such as job pre-requisites, command arguments, and compute requirements. After checking prerequisite conditions have been met, job(s) are submitted and monitored until completion when step-specific QC is performed with the exact command recorded in both the tracking database (**Fig S1**) and the detailed log file. The final task performed by the wrapper is the submission of the next analysis job, a process that continues until the final analysis step is completed.

The detection and recovery from both processing and hardware errors is also implemented in our system, using an approach similar to eHive (10)(Severin, et al., 2010) offering elegant solutions to address this. To achieve this level of automation the first potential bottleneck to overcome is the loading of relevant sample information into the tracking database; information such as sample type, sample metadata, and any sample connection information such as pedigree relationships or tumour/normal pairing. Correctly inputting this sample information is important as it ultimately determines the workflow selected; workflows that are fundamentally different for single samples, pedigree samples, and paired cancer samples for example (. The system works to detect both catastrophic errors (errors that cause the entire analysis to halt) and non-catastrophic errors (errors that do not cause analysis to halt but generate incorrect output). Non-catastrophic errors can be particularly problematic both in wasting ensuing CPU cycles and in making it difficult to pinpoint the exact point of failure in long running analysis.

Importantly, the system output is entirely reproducible, a feature which cannot be assumed in biological sciences as highlighted by a study discovering less than half of the microarray studies published in Nature Genetics were reproducible (11). To achieve reproducibility (a main goal of the GALAXY framework (12)), our system employs multiple tracking methods, an underlying mysql database and a detailed log file, with each method capable of independently reproducing previous results. While this design decision was initially implemented to ensure output consistency, it has proven useful in diagnosing pipeline crashes by offering two distinct reference points for pinpointing the exact point of failure. Full reproducibility requires more than detailing how commands were run, it requires the versioning of all files and binaries with all relevant version numbers recorded. This applies to the version of external binaries, the version of the larger code base, and the version of external annotation data sets. External annotation data in particular represents a challenge for reproducibility with rapid updates and changes to format typical of these external data sets. To manage such annotations consistently, all annotations are read from the original data source and stored locally in a standardized file format with standard file names. Annotation files from a single source are stored in time-stamped directories thus allowing the most up to date information to be automatically detected and utilized during analysis. This consistent handling allows for seamless updates as new annotations become available while additionally simplifying batch re-analysis when important annotation sets like dbSNP (13) are updated.

(McCoy, et al., 2013). This feature is particularly useful for maintaining up to date production pipelines that need to cope with frequent software updates and occasionally integrate new bioinformatics tools. This testing environment is utilized both in determining optimal parameters for individual algorithms but equally valuable in determining optimal software combinations by running permutations of discrete analysis steps such as alignment and variant calling. While here we describe the default workflow (**Fig S2**), the system is flexible with regard to software selection thus providing a ready-made testing environment, a feature currently available with tools like Nestly (14). This testing environment can be utilized both in determining optimal parameters for individual algorithms but also in determining effective software combinations by testing aligners and variant callers together for example. Currently a selection of some of the most utilized aligners and variant callers are available to facilitate such analyses (bowtie2 (15), BWA (2), isaac (16), GATK (5), isaac_variant_caller (16), and SAMtools (3)).

To address the often-low concordance rates between variant callers (17) and variant calling pipelines (18) our system allows the use of multiple variant callers with options for taking forward either the union or the intersection of raw variant calls depending on project-specific circumstances, similar to the approach taken by BAYSIC (19). In general taking the union of variants gives higher false positive rates and lower false negative rates while taking the intersection of variants gives lower false positive rates and higher false negative rates.

In the default workflow, each individual lane from a sample is aligned to the human reference genome GRCh37 using the short read aligner BWA (2) with default parameters unless a non phred33 base quality encoding is detected. Multiply aligned reads are removed from the SAM file (identified by the XT:A:R tag) and a sorted BAM file is subsequently generated and indexed. Alignment statistics are generated from the BAM file using the SAMtools (Li et al. 2009)flagstat command and only lanes with at least 90% of total reads aligning to the reference genome processed further. When all sequencing lanes from a sample have been aligned and passed quality control, a single merged BAM files is created using SAMtools merge. Duplicate reads are subsequently removed from the BAM file using the SAMtools rmdup command, a step that aims to avoid false positive variants arising due to PCR duplication, a process that often occurs during the library preparation step (20). Variant detection utilizes either a SAMtools/bcftools workflow (4) or GATK workflow (5) to generate a vcf file for each chromosome. To obtain high quality variants, a minimum variant quality score of 40 is applied with these variants subsequently divided based on chromosome and variant type (SNV or InDel) to facilitate timely processing. These variants are next compared for overlaps with EnsEMBL canonical transcripts (v75; overlap with coding exons or splice site) and exon variants run through the variant effect predictor (VEP) (7) to determine whether the variants represent non-synonymous changes and to obtain additional annotations information such as GMAF frequencies, common gene names, protein domains, GO terms, etc. Pre-calculated PolyPhen2 (8) , SIFT (9) and CADD (21) scores are also extracted from VEP to prioritize variants by predicting the functional impact of the missense mutations on the protein. Variants are overlapped with dbSNP (13) to determine whether the variant has been previously reported in the general population; resulting in the variant set being classified as novel, rare (<2% population frequency), common (>=2% population frequency), or unknown (no frequency available). Lastly, all information is aggregated and prioritized variant reports generated, such that variants most likely to have relevance to any disease phenotype are prioritized. During processing, data is archived, keeping raw sequence reads (FASTQ files), aligned reads (BAM file) and resultant variant calls (vcf file), along with tabulated metadata, making the file-based archive independent of the tracking database and ensuring reproducibility.

**Reference**s:

1. Gafni E, Luquette LJ, Lancaster AK, Hawkins JB, Jung JY, Souilmi Y, et al. COSMOS: Python library for massively parallel workflows. Bioinformatics. 2014.

2. Li H, Durbin R. Fast and accurate short read alignment with Burrows-Wheeler transform. Bioinformatics. 2009;25(14):1754-60.

3. Li H, Handsaker B, Wysoker A, Fennell T, Ruan J, Homer N, et al. The Sequence Alignment/Map format and SAMtools. Bioinformatics. 2009;25(16):2078-9.

4. Li H. A statistical framework for SNP calling, mutation discovery, association mapping and population genetical parameter estimation from sequencing data. Bioinformatics. 2011;27(21):2987-93.

5. McKenna A, Hanna M, Banks E, Sivachenko A, Cibulskis K, Kernytsky A, et al. The Genome Analysis Toolkit: a MapReduce framework for analyzing next-generation DNA sequencing data. Genome research. 2010;20(9):1297-303.

6. Taylor RC. An overview of the Hadoop/MapReduce/HBase framework and its current applications in bioinformatics. BMC bioinformatics. 2010;11 Suppl 12:S1.

7. McLaren W, Pritchard B, Rios D, Chen Y, Flicek P, Cunningham F. Deriving the consequences of genomic variants with the Ensembl API and SNP Effect Predictor. Bioinformatics. 2010;26(16):2069-70.

8. Adzhubei IA, Schmidt S, Peshkin L, Ramensky VE, Gerasimova A, Bork P, et al. A method and server for predicting damaging missense mutations. Nature methods. 2010;7(4):248-9.

9. Kumar P, Henikoff S, Ng PC. Predicting the effects of coding non-synonymous variants on protein function using the SIFT algorithm. Nature protocols. 2009;4(7):1073-81.

10. Severin J, Beal K, Vilella AJ, Fitzgerald S, Schuster M, Gordon L, et al. eHive: an artificial intelligence workflow system for genomic analysis. BMC bioinformatics. 2010;11:240.

11. Ioannidis JP, Allison DB, Ball CA, Coulibaly I, Cui X, Culhane AC, et al. Repeatability of published microarray gene expression analyses. Nature genetics. 2009;41(2):149-55.

12. Goecks J, Nekrutenko A, Taylor J, Galaxy T. Galaxy: a comprehensive approach for supporting accessible, reproducible, and transparent computational research in the life sciences. Genome biology. 2010;11(8):R86.

13. Sherry ST, Ward MH, Kholodov M, Baker J, Phan L, Smigielski EM, et al. dbSNP: the NCBI database of genetic variation. Nucleic acids research. 2001;29(1):308-11.

14. McCoy CO, Gallagher A, Hoffman NG, Matsen FA. Nestly--a framework for running software with nested parameter choices and aggregating results. Bioinformatics. 2013;29(3):387-8.

15. Langmead B, Salzberg SL. Fast gapped-read alignment with Bowtie 2. Nature methods. 2012;9(4):357-9.

16. Raczy C, Petrovski R, Saunders CT, Chorny I, Kruglyak S, Margulies EH, et al. Isaac: ultra-fast whole-genome secondary analysis on Illumina sequencing platforms. Bioinformatics. 2013;29(16):2041-3.

17. Liu X, Han S, Wang Z, Gelernter J, Yang BZ. Variant callers for next-generation sequencing data: a comparison study. PloS one. 2013;8(9):e75619.

18. O'Rawe J, Jiang T, Sun G, Wu Y, Wang W, Hu J, et al. Low concordance of multiple variant-calling pipelines: practical implications for exome and genome sequencing. Genome medicine. 2013;5(3):28.

19. Cantarel BL, Weaver D, McNeill N, Zhang J, Mackey AJ, Reese J. BAYSIC: a Bayesian method for combining sets of genome variants with improved specificity and sensitivity. BMC bioinformatics. 2014;15:104.

20. Kozarewa I, Turner DJ. Amplification-free library preparation for paired-end Illumina sequencing. Methods in molecular biology. 2011;733:257-66.

21. Kircher M, Witten DM, Jain P, O'Roak BJ, Cooper GM, Shendure J. A general framework for estimating the relative pathogenicity of human genetic variants. Nature genetics. 2014;46(3):310-5.
