# Supplementary material for: Reliably Detecting Clinically Important Variants Requires Both Combined Variant Calls and Optimized Filtering Strategies
Source: PLoS One. 2015 Nov 23;10(11):e0143199. doi: 10.1371/journal.pone.0143199 (PMC4658170; doi:10.1371/journal.pone.0143199)
Supplement: S1 Table — All short read aligners and variant callers commands utilized in our example. The commands listed match the exact commands run with the exception of the shortening of file names. The commands were chosen by either following documentation suggestions, or else by using default options. (DOCX) [file pone.0143199.s004.docx]

**S1 Table. Short read aligner and variant caller versions and commands.**

| **Software** | **Version** | **Commands** |
| --- | --- | --- |
| bowtie2 | 2.2.3 | 1) bowtie2 –p 16 -1 fq1 -2 fq2 –x hg19 \| samtools view –bt hg19.fa.fai - \| samtools sort - > bt2.bam |
| bwa | 0.7.10 | 1) bwa aln –t 16 hg19 fq1 > r1.sai  2) bwa aln –t 16 hg19 fq2 > r2.sai  3) bwa sampe hg19 r1.sai r2.sai fq1 fq2 \| samtools view –bt hg19.fa.fai - \| samtools sort - > bwa.bam |
| isaac aligner | 01.14.08.28 | 1) isaac-align -r sorted_reference.xml --base-calls-format fastq-gz –j 16 –m 40 –keep-aligned back –realign-gaps yes -o isaac_align –b readdir |
| GATK (raw) | 3.2.2 | 1) java -jar GenomeAnalysisTK.jar -T UnifiedGenotyper -I bwa.bam -o bwa_gatk.vcf -R hg19.fa –glm BOTH -metrics bwa_gatk.metrics -rf MappingQuality -mmq 10 |
| GATK (VQSR) | 3.2.2 | 1) java -jar GenomeAnalysisTK.jar -T VariantRecalibrator -R hg19.fa -input bwa_gatk.vcf -recalFile bwa_gatk.snv.recal -tranchesFile bwa_gatk.snv.tranches -resource:hapmap,known=false,training=true,truth=true,prior=15.0 hapmap_3.3.hg19.sites.vcf -resource:omni,known=false,training=true,truth=true,prior=12.0 1000G_omni2.5.hg19.sites.vcf -resource:1000G,known=false,training=true,truth=false,prior=10.0 1000G_phase1.snps.high_confidence.hg19.sites.vcf -resource:dbsnp,known=true,training=false,truth=false,prior=2.0 dbsnp_138.hg19.vcf -an QD -an MQ -an MQRankSum -an ReadPosRankSum -an FS -an DP -mode SNP  2) java -jar GenomeAnalysisTK.jar -T ApplyRecalibration -R hg19.fa -input bwa_gatk.def.vcf -tranchesFile bwa_gatk.snv.tranches -recalFile bwa_gatk.snv.recal -o bwa_gatk.vqsr.snv.vcf --ts_filter_level 99.5 -mode SNP  3) java -jar GenomeAnalysisTK.jar -T VariantRecalibrator -R hg19.fa -input bwa_gatk.vcf -recalFile bwa_gatk.indel.recal -tranchesFile bwa_gatk.indel.tranches -resource:mills,known=false,training=true,truth=true,prior=12.0 Mills_and_1000G_gold_standard.indels.hg19.sites.vcf -resource:dbsnp,known=true,training=false,truth=false,prior=2.0 dbsnp_138.hg19.vcf --maxGaussians 4 -an QD -an MQ -an MQRankSum -an ReadPosRankSum -an FS -an DP -mode INDEL  4) java -jar GenomeAnalysisTK.jar -T ApplyRecalibration -R hg19.fa -input bwa_gatk.def.vcf -tranchesFile bwa_gatk.indel.tranches -recalFile bwa_gatk.indel.recal -o bwa_gatk.vqsr.indel.vcf --ts_filter_level 99.0 -mode INDEL |
| isaac variant caller (raw) | 1.0.6 | 1) configureWorkflow.pl –bam bwa.bam –ref hg19.fa --config config.ini  2) make –j 16 |
| Isaac variant caller (No LowGQX variants) | 1.0.6 | 1) grep -v LowGQX bwa_isaac.vcf > bwa_isaac.noLowGQX.vcf |
| Samtools (no BAQ filtering) | 0.1.18 | 1) samtools mpileup -C50 -uDBf hg19.fa bwa.bam \| bcftools view -vcg - > bwa_samools.noBAQ.vcf |
| Samtools (BAQ filtering) | 0.1.18 | 1) samtools mpileup -C50 -uDf hg19.fa bwa.bam \| bcftools view -vcg - > bwa_samools.vcf |

All short read aligners and variant callers commands utilized in our example. The commands listed match the exact commands run with the exception of the shortening of file names. The commands were chosen by either following documentation suggestions, or else by using default options.
