# Supplementary material for: Reliably Detecting Clinically Important Variants Requires Both Combined Variant Calls and Optimized Filtering Strategies
Source: PLoS One. 2015 Nov 23;10(11):e0143199. doi: 10.1371/journal.pone.0143199 (PMC4658170; doi:10.1371/journal.pone.0143199)
Supplement: S2 Table — SNV stats from all eighteen possible software combinations derived from the pairing of the three aligners with each of the three variant callers run both with and without filtering. SNVs were overlapped to GIAB SNVs and false positive and false negative rates calculated. (DOCX) [file pone.0143199.s005.docx]

**S2 Table. SNV call overlaps with GIAB.**

| **Aligner** | **Variant Caller** | **Total SNVs** | **False Positive %** | **False Negative %** |
| --- | --- | --- | --- | --- |
| Bowtie2 | GATK (raw) | 224436 | 6.99 | 3.13 |
| Bowtie2 | GATK (filtered) | 223085 | 6.66 | 3.92 |
| Bowtie2 | isaac (raw) | 159982 | 5.97 | 5.30 |
| Bowtie2 | Isaac (filtered) | 74316 | 3.99 | 8.84 |
| Bowtie2 | Samtools (raw) | 166722 | 5.95 | 3.97 |
| Bowtie2 | Samtools (filtered) | 105900 | 3.76 | 6.73 |
| BWA | GATK (raw) | 225546 | 6.87 | 2.94 |
| BWA | GATK (filtered) | 224671 | 6.62 | 3.29 |
| BWA | isaac (raw) | 166528 | 6.31 | 4.41 |
| BWA | isaac (filtered) | 78002 | 4.51 | 7.93 |
| BWA | Samtools (raw) | 167295 | 5.96 | 6.39 |
| BWA | Samtools (filtered) | 106322 | 3.78 | 3.82 |
| isaac_ | GATK (raw) | 182928 | 3.97 | 3.71 |
| isaac | GATK (filtered) | 182703 | 3.92 | 3.29 |
| isaac | isaac (raw) | 129105 | 3.45 | 3.54 |
| isaac | isaac (filtered) | 70499 | 3.6 | 5.03 |
| isaac | Samtools (raw) | 120000 | 3.59 | 9.34 |
| isaac | Samtools (filtered) | 82164 | 2.96 | 9.87 |

Snv stats from all eighteen possible software combinations derived from the pairing of the three aligners with each of the three variant callers run both with and without filtering. SNVs were overlapped to GIAB SNVs and false positive and false negative rates calculated.
