# Supplementary material for: Reliably Detecting Clinically Important Variants Requires Both Combined Variant Calls and Optimized Filtering Strategies
Source: PLoS One. 2015 Nov 23;10(11):e0143199. doi: 10.1371/journal.pone.0143199 (PMC4658170; doi:10.1371/journal.pone.0143199)
Supplement: S3 Table — Deletion stats from all eighteen possible software combinations derived from the pairing of the three aligners with each of the three variant callers run both with and without filtering. Deletions were overlapped to GIAB deletions and false positive and false negative rates calculated. (DOCX) [file pone.0143199.s006.docx]

**S3 Table. Deletion call overlaps with GIAB.**

| **Aligner** | **Variant Caller** | **Total Deletions** | **False Positive %** | **False Negative %** |
| --- | --- | --- | --- | --- |
| Bowtie2 | GATK (raw) | 1997 | 27.14 | 28.81 |
| Bowtie2 | GATK (filtered) | 1934 | 25.49 | 30.51 |
| Bowtie2 | isaac (raw) | 2829 | 24.14 | 23.73 |
| Bowtie2 | Isaac (filtered) | 1471 | 29.23 | 38.98 |
| Bowtie2 | Samtools (raw) | 2565 | 26.47 | 28.81 |
| Bowtie2 | Samtools (filtered) | 2594 | 26.83 | 27.12 |
| BWA | GATK (raw) | 1918 | 25.18 | 28.81 |
| BWA | GATK (filtered) | 1862 | 24.01 | 30.51 |
| BWA | isaac (raw) | 2680 | 22.61 | 23.73 |
| BWA | Isaac (filtered) | 1376 | 26.38 | 40.68 |
| BWA | Samtools (raw) | 2657 | 25.63 | 27.12 |
| BWA | Samtools (filtered) | 2753 | 26.7 | 27.12 |
| isaac | GATK (raw) | 1615 | 19.81 | 28.81 |
| isaac | GATK (filtered) | 1603 | 19.65 | 33.90 |
| isaac | isaac (raw) | 2223 | 20.69 | 27.12 |
| isaac | Isaac (filtered) | 1156 | 23.36 | 37.29 |
| isaac | Samtools (raw) | 2356 | 23.26 | 30.51 |
| isaac | Samtools (filtered) | 2390 | 23.77 | 30.51 |

Deletion stats from all eighteen possible software combinations derived from the pairing of the three aligners with each of the three variant callers run both with and without filtering. Deletions were overlapped to GIAB deletions and false positive and false negative rates calculated.
