# Supplementary material for: Reliably Detecting Clinically Important Variants Requires Both Combined Variant Calls and Optimized Filtering Strategies
Source: PLoS One. 2015 Nov 23;10(11):e0143199. doi: 10.1371/journal.pone.0143199 (PMC4658170; doi:10.1371/journal.pone.0143199)
Supplement: S4 Table — Insertion stats from all eighteen possible software combinations derived from the pairing of the three aligners with each of the three variant callers run both with and without filtering. Insertions were overlapped to GIAB insertions and false positive and false negative rates calculated. (DOCX) [file pone.0143199.s007.docx]

**S4 Table. Insertion call overlaps with GIAB.**

| **Aligner** | **Variant Caller** | **Total Insertion** | **False Positive %** | **False Negative %** |
| --- | --- | --- | --- | --- |
| Bowtie2 | GATK (raw) | 2114 | 20.1 | 40.30 |
| Bowtie2 | GATK (filtered) | 2052 | 18.81 | 40.30 |
| Bowtie2 | isaac (raw) | 3245 | 18.43 | 31.34 |
| Bowtie2 | Isaac (filtered) | 1577 | 22.76 | 43.28 |
| Bowtie2 | Samtools (raw) | 3678 | 23.65 | 26.87 |
| Bowtie2 | Samtools (filtered) | 3793 | 25.07 | 25.37 |
| BWA | GATK (raw) | 1936 | 17.3 | 40.30 |
| BWA | GATK (filtered) | 1901 | 16.31 | 41.79 |
| BWA | isaac (raw) | 2985 | 16.65 | 34.33 |
| BWA | Isaac (filtered) | 1447 | 18.87 | 43.28 |
| BWA | Samtools (raw) | 3451 | 18.78 | 35.82 |
| BWA | Samtools (filtered) | 3553 | 19.36 | 35.82 |
| isaac | GATK (raw) | 1718 | 15.02 | 37.31 |
| isaac | GATK (filtered) | 1702 | 14.92 | 38.81 |
| isaac | isaac (raw) | 2542 | 15.03 | 31.34 |
| isaac | Isaac (filtered) | 1284 | 15.73 | 37.31 |
| isaac | Samtools (raw) | 3796 | 31.72 | 32.84 |
| isaac | Samtools (filtered) | 3826 | 31.94 | 32.84 |

Insertion stats from all eighteen possible software combinations derived from the pairing of the three aligners with each of the three variant callers run both with and without filtering. Insertions were overlapped to GIAB insertions and false positive and false negative rates calculated.
